# Tissue-specific insulin signaling mediates female sexual attractiveness

**DOI:** 10.1371/journal.pgen.1006935

**Published:** 2017-08-17

**Authors:** Tatyana Y. Fedina, Devin Arbuthnott, Howard D. Rundle, Daniel E. L. Promislow, Scott D. Pletcher

**Affiliations:** 1 Department of Molecular and Integrative Physiology, University of Michigan, 109 Zina Pitcher Pl, Ann Arbor, MI, United States of America; 2 Department of Zoology, University of British Columbia, Vancouver, B.C., Canada; 3 Department of Biology, University of Ottawa, 30 Marie Curie, Ottawa, ON, Canada; 4 Department of Pathology, University of Washington, Seattle WA, United States of America; 5 Department of Biology, University of Washington, University of Washington, Seattle, WA, United States of America; 6 Geriatrics Center, University of Michigan, 109 Zina Pitcher Pl, Ann Arbor, United States of America; Cornell University, UNITED STATES

## Abstract

Individuals choose their mates so as to maximize reproductive success, and one important component of this choice is assessment of traits reflecting mate quality. Little is known about why specific traits are used for mate quality assessment nor about how they reflect it. We have previously shown that global manipulation of insulin signaling, a nutrient-sensing pathway governing investment in survival versus reproduction, affects female sexual attractiveness in the fruit fly, *Drosophila melanogaster*. Here we demonstrate that these effects on attractiveness derive from insulin signaling in the fat body and ovarian follicle cells, whose signals are integrated by pheromone-producing cells called oenocytes. Functional ovaries were required for global insulin signaling effects on attractiveness, and manipulations of insulin signaling specifically in late follicle cells recapitulated effects of global manipulations. Interestingly, modulation of insulin signaling in the fat body produced opposite effects on attractiveness, suggesting a competitive relationship with the ovary. Furthermore, all investigated tissue-specific insulin signaling manipulations that changed attractiveness also changed fecundity in the corresponding direction, pointing to insulin pathway activity as a reliable link between fecundity and attractiveness cues. The cues themselves, cuticular hydrocarbons, responded distinctly to fat body and follicle cell manipulations, indicating independent readouts of the pathway activity from these two tissues. Thus, here we describe a system in which female attractiveness results from an apparent connection between attractiveness cues and an organismal state of high fecundity, both of which are created by lowered insulin signaling in the fat body and increased insulin signaling in late follicle cells.

## Author summary

Animals choose their mates based on specific attractive traits, like song and plumage characteristics in birds, or smell and call features in mammals. These traits often reflect the beneficial qualities of their bearer, including parasite resistance, parental care, and fecundity, all of which increase the success of a chooser in siring more and healthier progeny. Why certain traits rather than others are used in assessing the mate quality is not clear. In this article, we show that insulin signaling–a conserved nutrient-sensing pathway that affects female metabolic status—acts specifically in certain tissues to increase fecundity and, at the same time, increases attractiveness of female pheromones in the fruitfly, *Drosophila melanogaster*. Our discoveries support the idea that reliability in mate assessment depends on the activity of an underlying molecular pathway that affects both attractiveness and fecundity. This work provides a foundation for investigating the mechanistic links between an attractive trait (pheromones) and evolutionary fitness (egg production).

## Introduction

The quality and reproductive potential of a chosen mate is one of the most consequential decisions affecting an animal’s fitness. Mate choice is based on select external traits that determine attractiveness. Among these, indicator traits are assumed to reflect an underlying quality, although in some cases individuals present false signals to secure more or higher quality mates [1,2]. Despite extensive theoretical and empirical treatment of attractiveness and mate choice [3–5] it is not entirely clear why certain traits rather than others are used for mate quality assessment. Arguably one reason for this uncertainty is that most studies of mate choice rely on phenotypic correlations between attractiveness traits and fitness traits and forego mechanistic connections between them [6,7]. Thus, in insects, more fecund females tend to be more attractive to males [4,8,9]. However, it is generally unknown how attractive traits are linked to fecundity; whether females advertise their fecundity, allowing the potential for cheating to secure a better mate [10]; or whether males have evolved to evaluate honest (uncheatable) indicators of fecundity that are hardwired to a general physiological state. We would expect less reliability in mate quality assessment and greater opportunity for cheating when molecular pathways that determine fecundity are independent of those that influence attractiveness.

Recently, we have shown that global manipulation of insulin signaling, a central nutrient-sensing pathway in many animals, affects the composition and attractiveness of the female pheromone profile in the fruit fly, *Drosophila melanogaster*. This likely involves transcriptional regulation of genes that encode enzymes that synthesize cuticular hydrocarbons [11]. Insulin signaling also influences attractiveness of the male dung beetle, although it appears to do so during development through its influence on the overall size of sexually selected traits [12]. Nutrition and insulin signaling strongly influence both survival and reproductive output [13,14]. Nutrient restriction and reduced insulin signaling extend lifespan [15], but they also significantly reduce reproductive output, resulting in compromised fitness in competitive assays [16]. These considerations suggest the possibility that flies and beetles have evolved to “read” insulin pathway activity in different forms as an honest and accurate metric of reproductive potential. Do such signals derive directly from reproductive tissues, thus reflecting its current activity, or are they comprised of signals from several tissues, thus being more representative of global organismal state that is conducive to fecundity? In this manuscript, we begin dissecting mechanistic links between insulin signaling, reproduction, and attractiveness in *Drosophila*, by investigating tissue specificity of insulin signaling effects.

## Results

We reasoned that if a specific tissue is involved in the production and/or faithful transduction of a reliable insulin-dependent attractiveness cue, then modulation of insulin signaling in such a tissue should recapitulate the effects on attractiveness that we previously observed with global IS manipulations [11]. Several tissues are known to be intimately involved in *Drosophila* reproduction and attractiveness phenotypes, including the oenocytes (the cells involved in metabolic homeostasis and production of cuticular hydrocarbons), the fat body (a primary endocrine and nutrient storage tissue), and ovarian cells themselves [17–19].

**Fig 1:**
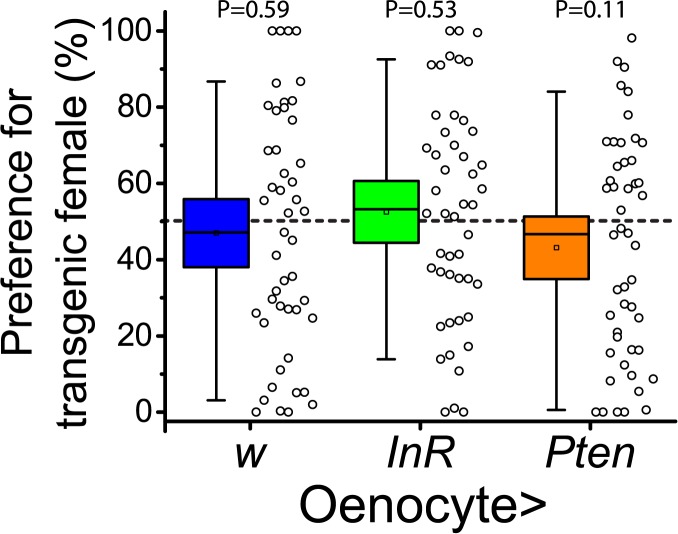
Insulin signaling in oenocytes does not affect attractiveness. An oenocyte-specific geneswitch Gal4 driver, *PromE800G*.*S*.*-Gal4*, was used to manipulate insulin signaling in these cells by causing RU486-dependent expression of insulin receptor (InR) for activation or of the phosphatase Pten for inhibition. The driver crossed to a standard laboratory strain (w^-^) served as a control. Two-choice preference trials were then performed comparing females fed RU486 to those fed vehicle. P-values from Wilcoxon tests are reported. Box plot boundaries here and in other plots represent 99% confidence intervals around the mean.

We first interrogated oenocytes, which produce attractiveness cues. These subcutaneous abdominal cells manufacture cuticular hydrocarbons (CHCs) that are transported to the outside cuticle to aid in desiccation resistance and social communication. In addition, oenocytes have often been compared to mammalian hepatocytes because of their metabolic functions and interactions with the fat body [17,18,20]. Such a dual internal-external function makes these cells strong candidates for revealing metabolic status to the outside world. Surprisingly, using two independent transgenic methods to induce oenocyte-specific activation of IS (through overexpression of the insulin receptor, *InR*) and inhibition of IS (through overexpression of the phosphatase *Pten*) in adult females we observed no subsequent effect on attractiveness (Figs 1 and S1). The absence of oenocyte cell-autonomous IS effects suggests that these cells translate non-insulin signals that reflect IS status in other tissues into attractiveness-relevant CHC changes.

**Fig 2:**
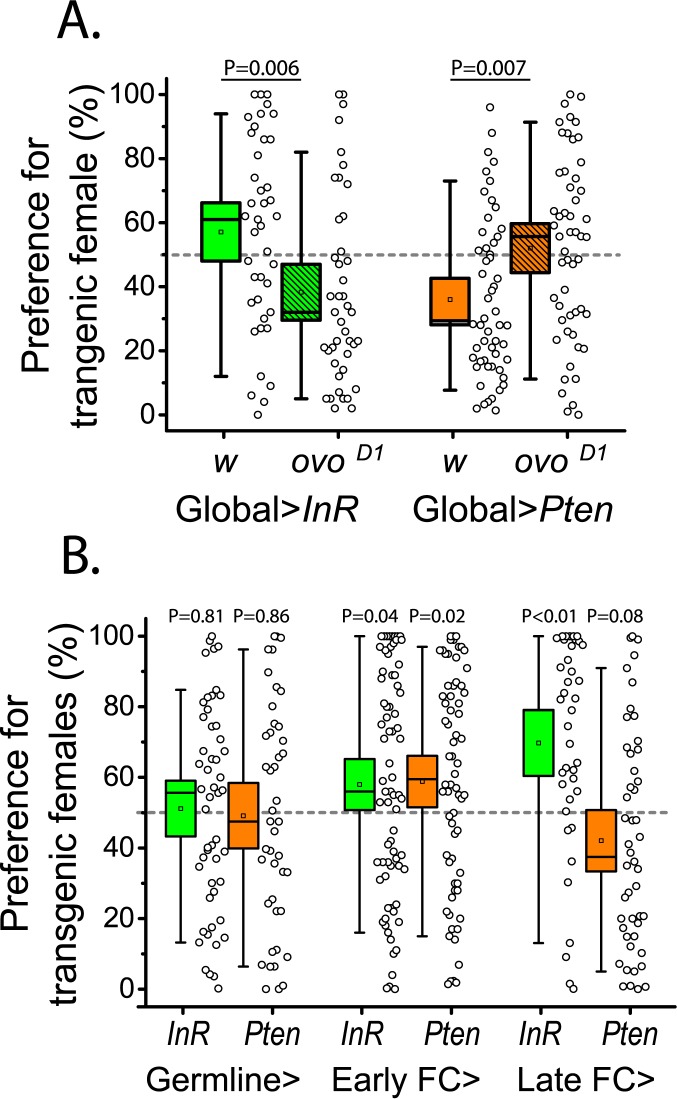
Ovarian function influences insulin-dependent attractiveness. (A) Removal of vitellogenic ovaries reverses attractiveness of flies with globally manipulated insulin signaling. Introducing a single copy of *ovo*^*D1*^ into transgenic flies blocks egg development at early previtellogenic stages, and in flies with global (*gsTub5-Gal4*) insulin signaling manipulations, it reverses the effects on attractiveness. (B) Manipulation of insulin signaling in late follicle cells (*C204-Gal4*,*TubP-Gal80*^*ts*^) is sufficient to affect attractiveness. Opposing insulin signaling manipulations in the germline (Nos-Gal4,TubP-Gal80^ts^, first panel) or early follicle cells (C587-Gal4, TubP-Gal80^ts^, middle panel) did not produce opposing effects on attractiveness, while manipulations in late follicle cells recapitulated effects of global insulin signaling manipulations (right panel).

Because manipulations of IS in oenocytes did not affect attractiveness, we next sought to identify other tissues that may be involved. Female reproductive output is one of the primary fitness components of a choosing male, and therefore the determination of reliable attractiveness indicators is expected to either originate in reproductive tissues or be modulated by them. To determine whether functional ovaries are required for changes in attractiveness generated by global manipulations in insulin signaling, we combined the *ovo*^*D1*^ mutation, which blocks oogenesis at previtellogenic stages and results in rudimentary ovaries with poorly differentiated germline cells [21], with the transgenic constructs used to manipulate insulin signaling. We found that elimination of vitellogenic ovaries reversed the effects of both global IS activation and inhibition on female attractiveness (Fig 2A).

To determine whether manipulation of IS in the reproductive system is sufficient to modulate attractiveness, we focused on two ovarian tissues, the germline and the somatic follicle cells. In the germline, insulin receptor is required for stem cell proliferation and progression to vitellogenesis [22,23]. Surprisingly, our germline manipulations of IS did not significantly affect female attractiveness (Fig 2B). Follicle cells surrounding early egg chambers undergo replicative divisions until stage 7–8 [24], after which they stop proliferating, enter an endoreplication cycle, and begin yolking the oocyte. These developmental steps proceed normally in the absence of insulin receptor, suggesting low insulin signaling during this period [23]. When IS was manipulated in undifferentiated early follicle cells, we failed to observe characteristic opposing changes in female attractiveness from opposing IS manipulations (Fig 2B); instead, attractiveness increased in response to both IS manipulations, perhaps, due to some non-specific response related to growth coordination. In contrast to germline and early follicle cells, in late follicle cells, which surround vitellogenic oocytes, IS upregulation promoted attractiveness while IS downregulation decreased it (Fig 2B).

**Fig 3:**
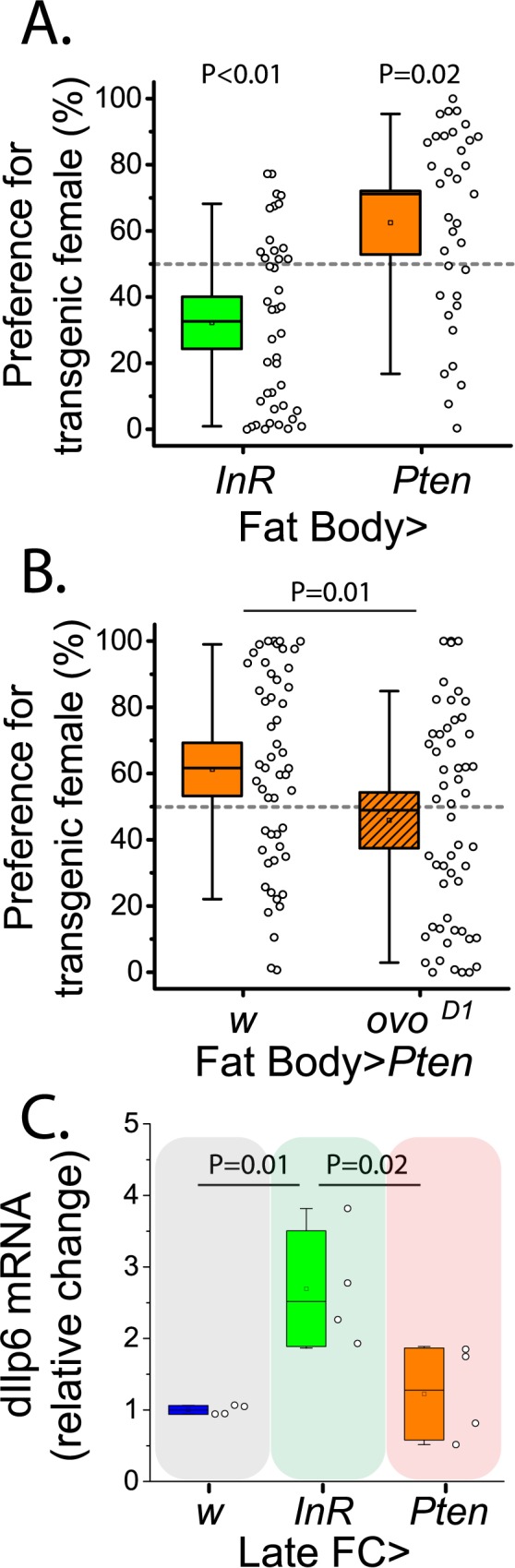
Attractiveness is opposed by fat body insulin signaling, and ovaries are required for the effect. (A) Female attractiveness is increased by downregulation of insulin signaling in the fat body (*Yolk-Gal4*), and decreased by upregulation of the pathway in this tissue. (B) The increased attractiveness of females with reduced insulin signaling in the fat body is abolished in females lacking vitellogenic ovaries due to *ovo*^*D1*^. (C) *Dilp6* gene expression in the fat body is significantly increased in females following upregulation of insulin signaling in late follicle cells with *C204-Gal4*,*TubP-Gal80*^*ts*^ (no difference found between control and females with downregulated IS, P = 0.86). Comparisons associated with increases in female attractiveness are indicated by green background shading, while those associated with decreased attractiveness are indicated by red background shading.

In *Drosophila*, the primary tissue involved in organism nutrient homeostasis is the fat body, which interacts closely with oenocytes, e.g. for lipid mobilization in response to starvation [18,20,25], and with the ovary, e.g. for yolking the oocytes [19]. Therefore, we next tested whether manipulations of IS targeted to the fat body affect attractiveness. Interestingly, activation of IS in adult fat body reduced female attractiveness, while inhibition potentiated it, which is opposite of what we observed for global and follicle cell manipulations (Fig 3A). Elimination of vitellogenic ovaries in flies with IS downregulated in the fat body abolished their increased attractiveness (Fig 3B).

It has been shown that starvation or direct FOXO activation in the fat body upregulates transcription of fat body *dIlp6*, which acts as a systemic antagonist of IS by decreasing *dilp2* transcription and release from the brain [26]. It was therefore possible that fat body IS manipulations influenced attractiveness through negative feedback control of the insulin producing cells (IPC) of the brain. However, we found that manipulations of IS in the IPC or modulation of their neural activity, as well as starvation (all of which result in changes in hemolymph Dilp2 levels, see [27]), failed to produce changes in attractiveness (S2A Fig). Interestingly, increased IS in the follicle cells surrounding vitellogenic oocytes resulted in upregulation of both *dIlp2* mRNA in the head and *dIlp6* mRNA in the fat body (Figs S2B & 3C), suggesting unique ovarian modulation of fat body and systemic metabolism.

**Fig 4:**
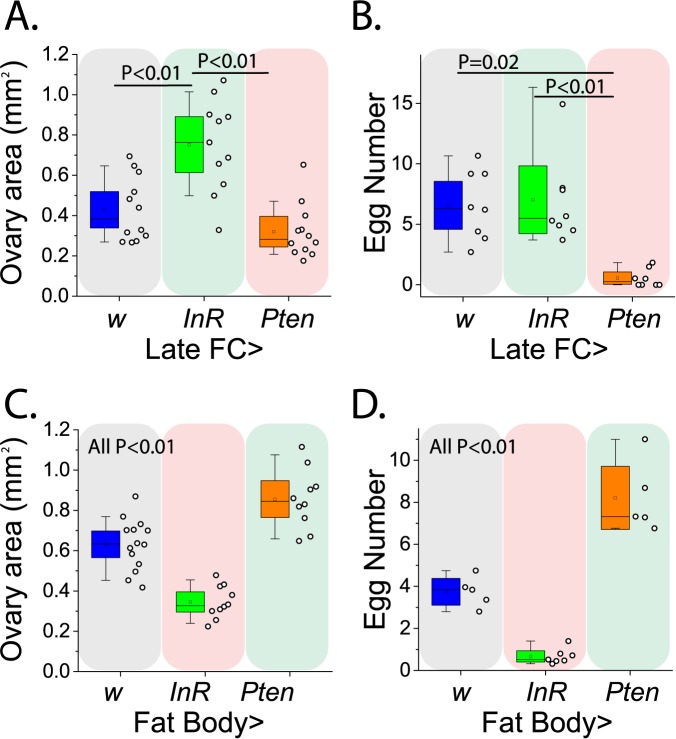
Female attractiveness predicts ovarian function. (A,B) Upregulation of IS in the late follicle cells surrounding vitellogenic oocytes (*C204-Gal4*,*TubP-Gal80*^*ts*^) increases female ovarian function and IS downregulation in these cells decreases ovarian function. Transgenic and control females differ in (A) virgin ovary size (ANOVA F = 17.88, P = 0.0001) and in (B) mated female fecundity (ANOVA F = 7.343, P = 0.0041). No difference detected between Late FC>*w vs Pten* in ovary size (P = 0.31), and between Late FC>*w vs InR* (P = 0.85). (C, D) Insulin signaling in the fat body (*Yolk-Gal4*) decreases female reproductive value: Transgenic and control females differ in both (C) virgin ovary size (F = 43.92, P<0.0001) and (D) mated female fecundity (F = 80.22, P<0.0001). Comparisons associated with increases in female attractiveness are indicated by green background shading, while those associated with decreased attractiveness are indicated by red background shading.

Reliable cues require a mechanistic link to fitness. We therefore asked whether the tissue-specific IS manipulations that increased attractiveness also increase reproductive potential by measuring ovary size in virgins and fecundity in females mated to Canton-S males. Consistent with our expectation, female ovarian function was increased by tissue-specific IS manipulations that promoted attractiveness, and it was decreased by IS manipulations that decreased attractiveness (Fig 4). Strikingly, when tissue-specific IS manipulations had opposite effects on attractiveness (e.g. manipulations in late follicle cells versus those in the fat body) they caused parallel and opposing effects on female ovarian function as well. Not unexpectedly, IS manipulations that did not change attractiveness had variable effects on fecundity (S3 Fig).

**Fig 5:**
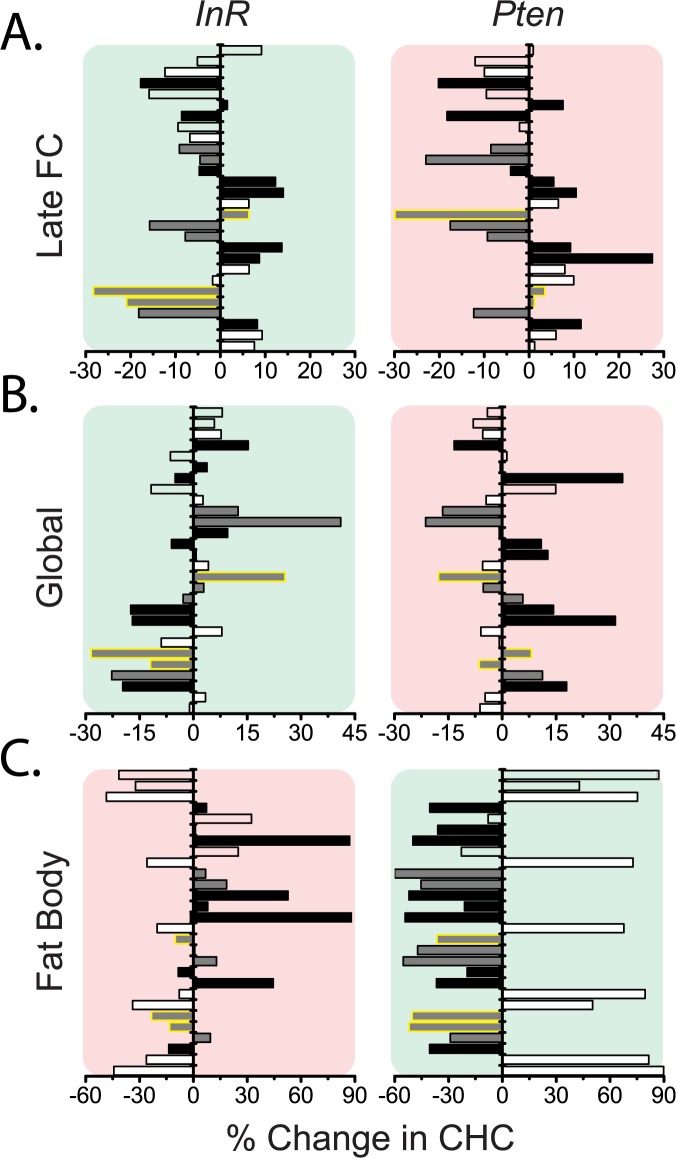
Cuticular hydrocarbon profiles are influenced by tissue-specific manipulations of insulin signaling. (A) Increased insulin signaling in late follicle cells (*C204*,*TubP-Gal80*^*ts*^*>InR*) induces changes in CHC composition that are distinct from those caused by decreases in insulin signaling (*C204*,*TubP-Gal80*^*ts*^ >*Pten)*. CHCs indicated by a yellow outline respond differently to the two manipulations. Comparisons associated with increases in female attractiveness are indicated by green background shading, while those associated with decreased attractiveness are indicated by red background shading. (B) CHC changes in response to global IS upregulation (gsTub5>*InR*) oppose those changes in response to downregulation of IS (gsTub5>*Pten*, Binomial sign test P = 0.0001 for 24 out of 28 CHCs showing opposite response). Saturated CHCs uniformly increased in response to the latter treatment (Binomial sign test P = 0.0078 for 7 out of 7 saturated CHCs responded in the same fashion). Similar changes in 5-T (5-C23:1), 7-T, and 5-P (5-C23:1) are observed for global fat body manipulations as for follicle cell manipulations. (C) Upregulation of insulin signaling in the fat body (Yolk>*InR*) decreased CHC saturation (P = 0.0078 for saturated CHCs), and the response for the whole CHC profile is reversed by opposing IS manipulation (Yolk>*Pten*; p = 0.0004 for 23/28 CHCs showing opposing response). CHC responses to fat body IS upregulation were very similar to CHC responses to global IS downregulation (P = 0.006 for 21/28 CHCs changing in the same direction). Similarly, fat body downregulation and global upregulation of IS produced unidirectional changes for 18 out of 28 CHCs (p = 0.092). Individual CHCs are presented in the following order (from bottom to top): nC21; C22:0; 7,11-TD; 9-C23:1; 7-T, 5-C23:1(5-T); nC23; C24:0; 9,13-C25:2; 7,11-PD; 9-P; 7-P; 5-C25:1; nC25; 9,13-HD; 7,11-HD; 5,9-HD; 7-H; 5-H; nC27; unknown #1; 9,13-ND; 7,11-ND; 29MeBr; 5,9-ND; nC29; unknown #2; unknown #3. Changes in each CHC are color coded in accordance with the degree of CHC saturation: white bars represent saturated CHCs, dark grey bars represent CHCs with single double bond (monoenes), and black bars represent CHCs with two double bonds (dienes). Bars representing the three unidentified CHCs, and a single 29MeBr are empty.

How is it that internal changes in insulin signaling and reproductive effort are reflected externally and detected by males as reliable cues? To investigate this question, we analyzed cuticular hydrocarbon (CHC) profiles comprised of 28 CHCs ranging in length from 21 to 29 carbons. Several of these CHCs have been shown to serve as important pheromonal cues in *Drosophila* [28]. Intriguingly, the significant effects on attractiveness of IS in late follicle cells appear to have resulted from changes in a few CHCs (Fig 5A, highlighted in yellow). 5-T and 7-T abundances were decreased by insulin signaling, and attractiveness was negatively associated with the levels of these CHCs. 5-P, on the other hand, exhibited the opposite effect: its abundance was increased by insulin signaling and positively associated with attractiveness. Notably, 5-T and 7-T are known courtship inhibitors [29,30], and 5-P decreased significantly following mating in female flies [31]. Females in which IS was globally manipulated experienced similar patterns of change in the CHCs that were affected by follicle cell IS activation (5-T, 7-T, and 5-P; Fig 5B). In addition, an increase in the abundance of saturated CHCs was noticeable following global IS upregulation; this pattern was reversed in unattractive females with global IS suppression (Fig 5B). Elimination of vitellogenic ovaries, which abolished the effects of global IS manipulations on attractiveness, also reversed changes in many CHCs, including the increase in saturated compounds, and removed the CHC signature of late-follicle-cell-specific IS manipulations (S4A Fig, compare to Fig 5B). CHC profiles were largely unaffected by germline IS manipulations, consistent with the absence of effects on attractiveness (S4B Fig).

We observed the most extensive changes in relative CHC abundance following fat body IS manipulations (Figs 5C and S6). Similar to the IS effects on attractiveness, CHC changes were largely opposite to those observed following global IS manipulations of the same type, and removal of vitellogenic ovaries reversed changes in saturated CHCs (S5A and S6 Figs). However, no characteristic pattern was apparent for changes in 5-T, 7-T, and 5-P. Interestingly, removal of vitellogenic ovary through the ovo^D1^ mutation in flies with global IS downregulation produced CHC profiles similar to those caused by IS downregulation in the fat body alone (compare right panels in Fig 5C and S4A Fig), suggesting that attractiveness is determined by an integration of effects in different tissues and that reproductive tissues predominate. Such a relationship could be mediated by fat body *dIlp6* transcription, which is upregulated by both increased ovarian IS and by decreased IS in the fat body. Although IS manipulations in the oenocytes had no effect on female attractiveness, we observed quite a few changes in CHC composition (S5B Fig). Unlike global or fat body IS manipulations, which were associated with largely uniform compositional shifts in both saturated and unsaturated CHCs, there was no such coordinated response to oenocyte IS manipulations, suggesting a qualitatively different effect of IS manipulation in oenocytes compared to tissues that signal attractiveness.

## Discussion

Individuals choose mates to maximize reproductive output, and our data indicate that at least one important component of mate choice in *D*. *melanogaster*, the attractiveness of pheromone profiles, is mechanistically linked to reproductive function. Our goal was to investigate the molecular nature of this link to, ultimately, understand the mechanism and the extent of reliability in *Drosophila* mate assessment. Global activation of insulin signaling is known to increase attractiveness in flies [11], and we found that this was unlikely to be due to systemic effects of brain insulin-producing cells. Instead, tissue-specific effects predominate through local modulation of the pathway. In the oenocytes, which produce attractiveness cues as cuticular hydrocarbons, IS did not influence attractiveness suggesting these cells receive non-insulin cues from other tissues. Late follicle cells may be one source of such cues as IS in these cells potentiated both attractiveness and reproductive function. The fat body may be another, although IS in this tissue reduced both traits. Vitellogenic ovaries were found to be necessary for global and fat body insulin signaling manipulations to influence attractiveness. Furthermore, manipulation of insulin signaling in germline cells or in early follicle cells produced inconsistent effects on attractiveness and reproductive output. Together, these results indicate that insulin-dependent attractiveness cues, produced by oenocytes and manifested as cuticular hydrocarbon profiles, are determined cell non-autonomously by integration of nutrient-sensing pathway activities from different tissues. Reproductive effort, reflected accurately in late-stage follicle cells, may therefore constitute one potential mechanism of honesty and reliability in this system of mate choice.

Insulin signaling in the fat body produced opposite effects on attractiveness compared to those caused by global and late follicle cell manipulations. One explanation for why insulin signaling in the fat body acts this way may involve its function in nutrient storage. Reduced insulin signaling in the fat body may promote nutrient release and/or limit nutrient uptake by inducing local insulin resistance. This would result in a net increase in hemolymph metabolites that are available to the ovaries, which may promote insulin signaling and other anabolic pathways in ovarian tissues, increase fecundity, and improve attractiveness. Such a reciprocal relationship between these two tissues is supported by our CHC data and *dilp6/dilp2* expression data (Figs 3C & S2A). Thus, decreased CHC desaturation in more fecund and more attractive females with downregulated fat body insulin signaling is ablated by removal of vitellogenic ovaries, suggesting that ovaries influence fat body IS and modify CHC saturation levels. Either increasing insulin sensitivity in the late ovarian follicle cells or decreasing insulin-sensitivity of the fat body benefited attractiveness and fecundity, perhaps via shifting the nutrient flux away from fat body and towards the eggs. Activation of insulin signaling in the late follicle cells also increased *dilp6* expression in the fat body, the latter being similar to what has been observed by others (along with decrease in systemic *dilp2* expression and release) in response to starvation [20]. The fact that both increasing IS in late follicle cells or decreasing it in the fat body resulted in increased brain *dilp2* expression suggests unique ovarian modulation of systemic IS, which is known to maintain germline stem cell proliferation rate [22,23].

We propose a model in which an attractiveness signal originates in the ovary following an increase in insulin signaling in late follicle cells. This signal, whether encoded by a secreted molecule or by a change in the metabolite composition of the hemolymph due to ovarian activation, signals to the fat body to increase its insulin resistance. With the diminished ability of the fat body to absorb nutrients essential for egg production and/or with increased release of such nutrients, a positive feedback loop is established with the ovary. Whether attractive CHC profiles in wild flies are determined solely by IS in ovarian late follicle cells (and, possibly, other ovarian tissues) or whether they are potentiated by an independent fat body metabolic signal remains to be determined. Our CHC profile and fecundity analyses following tissue-specific IS manipulations suggest the latter because fat body IS manipulations resulted in CHC changes distinct from those produced by follicle cell manipulations and because both tissue manipulations affected attractiveness and fecundity. Nevertheless, distinguishing between these two possibilities and identifying the molecule(s) that may encode signals between tissues are important avenues for future research.

What effector pathways play a role in this mechanism underlying reliable cues? The observed changes in the relative abundance of saturated CHCs upon insulin signaling manipulations indicate that an important desaturase enzyme, encoded by the gene *Desaturase 1 (Desat1)*, might be influential (see also [11]). *Desat1* is expressed in both the fat body and the oenocytes, and reduced *Desat1* expression results in increased abundance of saturated CHCs at the expense of unsaturated ones [32,33]. Of note, *Desat1* expression varies between tissues, particularly in response to nutrient demand, in both flies and mammals [20,34], while its vertebrate homolog, *SCD1*, is a key regulatory enzyme required in lipogenic tissues for fat storage [18,25]. We speculate that downregulation of *Desat1* is needed for lipid mobilization and release to growing oocytes, similar to what has been observed for starvation response [20]. It is conceivable, that males detect this “anti-desaturation” signal as a proxy for increased hemolymph nutrient levels, which is required for fecundity. Furthermore, changes in CHC profiles following insulin signaling manipulations in late follicle cells are distinct from those in the fat body, supporting our notion that both of these criteria (i.e. low insulin signaling in the fat body coupled with high insulin signaling in late follicle cells) indicate a high-nutrient, high fecundity state to males. This state is distinct from one characterized by general nutrient demand (i.e., low insulin signaling in both tissues) that would be expected to manifest during starvation.

Knowing the molecular links between animal attractiveness and reproductive potential is important for understanding evolutionary pressures acting on individual traits. If changes in CHC composition are a side effect of metabolic changes induced by reproductive effort or if females purposefully send honest signals reflecting their fecundity, then males would accurately “read” female fecundity from CHC profiles. If, however, the CHC profiles are free to be altered independently of fecundity, then cheating may evolve as less fecund females nevertheless seek to attract the best males [4,9]. Indeed, theoretical studies suggest that if a fitness signal or cue is honest or reliable on average then signaling or detection systems will be maintained in a population [10]. A recent comprehensive study confirms that *D*. *melanogaster* males consistently select more fecund females in laboratory conditions [8], suggesting that mate selection is based on either honest signals or reliable cues. We describe a system in which female attractiveness is produced by an organismal state of high fecundity and abundance of vitellogenic oocytes, which is in turn created by low insulin signaling in the fat body and high insulin signaling in late follicle cells. Notably, insulin signaling in these tissues is sensitive to environmental conditions, providing a mechanism through which information from the outside can be integrated with internal state. Future identification of the molecule(s) carrying attractiveness signals among tissues and understanding how they are influenced by internal and external perturbations will help reveal nature of sexual communication and stimulate new insights into how animals may evolve to cheat the system.

## Methods

### Fly stocks and husbandry

Canton-S and ovo^D1^ (BSC #1309, [21]) stocks were obtained from the Bloomington Stock Center. *Tublin5*-GeneSwitch flies were made by cloning the promoter of alphatubulin into the pSwitch2 vector. *UAS-Pten* (III) [35] and *UAS-InR* [36] overexpression lines were obtained from Dr. B. Edgar. *UAS-TrpA1* [37] and *UAS-Kir*_*2*.*1*_ [38] were obtained from Dr. P. Garrity and Dr. R. Baines, respectively, and the latter line was combined with *TubP-Gal80*^*ts*^ (BSC #7017) to bypass developmental effects. All these UAS lines were recently backcrossed for at least 7 generations to a standard *w*^*1118*^ (“*w*”) line that was used in control crosses. All tissue specific manipulations focused on expressing UAS transgenes only during the adult stage. Oenocyte drivers ‘+; *PromE800-Gal4*, *tubP-Gal80*^*ts*^; +’ [17] and ‘*w*;;*PromE800G*.*S*.*-Gal4*’ [20] were provided by Drs. J. Levine and H. Jasper respectively. Created in Dr. J. Hoffman lab [39] *Yolk-Gal4* driver naturally initiates expression in adult female fat body starting at 3–5 days of age when yolk synthesis commences. Germline and early follicle cell drivers ‘+*;Nanos-Gal4;TubP-Gal80*^*ts*^’ and ‘*C578-Gal4;+;TubP-Gal80*^*ts*^’ were obtained from Dr. Y. Yamashita. Late follicle cell driver *C204-Gal4* [40] was obtained from Bloomington Stock Center (#3751) and was combined with *TubP-Gal80*^*ts*^ (BSC #7017). Geneswitch Dilp2-Gal4 [41] and Dilp2R-Gal4 [42] lines were kindly provided by Dr. H. Jasper and Dr. E. Rulifson. Several of the above lines were also used to combine two transgenes within the same fly, including *Tublin5*-GeneSwitch and *UAS-Pten*, *Tublin5*-GeneSwitch and *UAS-InR*, *and Yolk-Gal4 and UAS-Pten*. For our experiments we used the following female progeny (with abbreviations): ‘*w*;;*PromE800G*.*S*.*-Gal4/w*’ (Oenocyte>*w*), ‘*w*;;*PromE800G*.*S*.*-Gal4/UAS-InR’* (Oenocyte>*InR*), ‘*w*;;*PromE800G*.*S*.*-Gal4/UAS-Pten’* (Oenocyte>*Pten*), ‘*w;;Tub5G*.*S*.*-Gal4*,*UAS-InR/w*’ (Global>*InR*, *w*), ‘*ovo*^*D1*^*/w;;Tub5G*.*S*.*-Gal4*,*UAS-InR/w*’ (Global>*InR*, *ovo*^*D1*^), ‘*w;;Tub5G*.*S*.*-Gal4*,*UAS-Pten/w*’ (Global>*InR*, *w*), ‘*ovo*^*D1*^*/w;;Tub5G*.*S*.*-Gal4*,*UAS-Pten/w*’ (Global>*InR*, *ovo*^*D1*^), ‘*w;Nanos-Gal4/w; TubP-Gal80*^*ts*^*/w’ (*Germline*>w)*, ‘*w;Nanos-Gal4/w;UAS-InR*,*TubP-Gal80*^*ts*^*/w’ (*Germline*>InR)*, ‘*w;Nanos-Gal4/w;UAS-Pten*,*TubP-Gal80*^*ts*^*/w’ (*Germline*>Pten)*, ‘*C587-Gal4/w*;;*TubP-Gal80*^*ts*^*/w*’ (Early FC>*w*),*)*, ‘*C587-Gal4/w*;;*UAS-InR*,*TubP-Gal80*^*ts*^*/w*’ (Early FC>*InR*), ‘*C587-Gal4/w*;;*UAS-Pten*,*TubP-Gal80*^*ts*^*/w*’ (Early FC>*Pten*),*)*, ‘*w*;;*C204-Gal4*,*TubP-Gal80*^*ts*^*/w*’ (Late FC>*w*),*)*, ‘*w*;; *C204-Gal4*,*UAS-InR*,*TubP-Gal80*^*ts*^*/w*’ (Late FC>*InR*), ‘*w*;; *C204-Gal4*,*UAS-Pten*,*TubP-Gal80*^*ts*^*/w*’ (Late FC>*Pten*), ‘*Yolk-Gal4/w;;*’(Fat Body>*w*), ‘*Yolk-Gal4/w;;UAS-InR/w*’(Fat Body>*InR*), ‘*Yolk-Gal4/w;;UAS-Pten/w*’(Fat Body>*Pten*), ‘*w;;UAS-InR/w*’ and *‘w;;UAS-Pten/w*’ as additional (UAS only) controls, ‘*Yolk-Gal4/w;;UAS-Pten/w*’(Fat Body>*Pten*, *w*), ‘*Yolk-Gal4/ovo*^*D1*^*;;UAS-Pten/w*’(Fat Body>*Pten*,*ovo*^*D1*^), ‘+; *PromE800-Gal4*, *tubP-Gal80*^*ts*^*/w*; +’(Oenocyte>*w*), ‘+; *PromE800-Gal4*, *tubP-Gal80*^*ts*^*/w*; *UAS-InR/w’*(Oenocyte>*InR*), ‘+; *PromE800-Gal4*, *tubP-Gal80*^*ts*^*/w*; *UAS-Pten/w’*(Oenocyte>*Pten*).

For all experiments, larvae were cultured in cornmeal-sugar-yeast “larval” media. Virgin adults were collected shortly after eclosion and maintained on 10% sugar/yeast (SY) food. Experimental flies carrying geneswitch Gal4 drivers were placed onto 10% SY food supplemented with RU486 (200 μM) to activate transgene expression (treatment) or with vehicle only (80% ethanol, control) for 10–15 days before experiments. Experimental flies carrying *Gal80ts*, were raised in 18°C, and transferred to 25°C upon eclosion for 3-7days before using in experiments (same age control as transgenic flies were used in each replicate for pairwise comparisons). Flies with Yolk-Gal4 driver were used as 6-7do. All other flies were maintained at 25°C and 60% relative humidity in a 12:12 h light:dark cycle. Fresh food was provided every 2 or 3 days. Detailed media recipes can be found in [43].

### Attractiveness assays

Video analysis was used to assess male choice between transgenic mutant and control females. In this assay, two-choice subject females (i.e., mutant and control) were decapitated and embedded in agar 15 mm apart and 7–10 mm away from the side of the dish. After the agar solidified, a single, 3–4 day-old Canton-S male (previously isolated for 24h) was aspirated in the arena and given 5–10 min to acclimate to the new environment. Video recording was then started, and it continued for 30 min. Videos were recorded at 2 frames per second and converted to AVI file format, which was analyzed with our VideoFly software. The software calculates the amount of time spent by the choosing fly inside a circle of 3 mm radius centered on each decapitated subject fly. Flies that did not court, identified as instances where the total time spent in the two target regions was less than 50 sec (2.8% of the total time of observation), were removed from further analysis. Male preference was calculated as the percentage of time males spent in the circles centered on transgenic (or otherwise manipulated) female divided by the total time spent in both circles. Detailed comparisons of the results from observed courtship assays and video assays confirmed that the latter accurately reflect male courtship behaviors [11, 45]. Using CHC transfer between live flies we have also previously shown that in these behavioral assays, female attractiveness is determined exclusively by pheromone profiles [11,44].

### Ovary size and fecundity measurements

For all experiments ovary size and fecundity was measured at the same female age as attractiveness. Ovaries were removed by dissection and placed in PBS on a slide, with a coverslip that was raised and supported by two pieces of tape to standardize thickness throughout. Digital pictures of the dissected ovaries were obtained using a Zeiss STEMI SV-11 microscope, and the images were analyzed using open source software ImageJ 1.x. The contour containing each ovary was outlined using the “freehand selections” tool, and the subsequent area of each contour was recorded in mm^2^. Two ovaries were examined for each female, and ovary size is reported as the average between the two. For fecundity measurement, females were first placed in groups with Canton-S males at 1:1 sex ratio for 2 days. The females were then isolated from males and placed in groups of 4–5 in standard *Drosophila* food vials containing 10% yeast and 10% sugar media for 2–3 days. Fecundity is expressed as number of eggs per female per day.

### CHC extraction and presentation

Cuticular hydrocarbons were extracted as previously described [11,44]. Each treatment was represented by 3–4 samples of 3–5 flies each. CHC samples were analyzed in the Rundle laboratory following their standard protocol [45]. CHC relative abundances were determined by dividing the area under each corresponding CHC peak by the area integrated for all 28 reliably identifiable peaks; these data are located in Supplemental File 1. Although the pattern of peaks was broadly consistent with those chemically identified by Foley et al. [46], the precise correspondence of three peaks was unclear. Because our purpose was to identify changes in CHC profiles upon IS manipulation, we have expressed, for each CHC, the percent increase or decrease in IS manipulated females relative to the mean abundance from control females.

### Quantitative PCR analysis

Trizol (Invitrogen) was used to extract total RNA from abdominal tissue (abdomens with ovaries and guts removed) of ~20 virgin females for dIlp6 expression analysis, or from 20–30 heads for dIlp2 expression analysis. The tissues were harvested from females of the same age as the ones used in attractiveness experiments. The extracted RNA was reverse transcribed into cDNA by Superscript III First-Strand Synthesis (Invitrogen) using oligo-dT primers. For each independent RNA extraction/cDNA reaction, 3 replicate RT-PCR reactions were performed using SYBR Green PCR Master Mix (Applied Biolsystems) with specific primers. The quantitative levels were normalized to an endogenous control *Tub5* or *RpL32*, as calculated by the ΔΔCT method [47], and results were presented as fold-change of transgene-expressing to control flies in expression levels. The following primers were used: *dilp6*F (CGATGTATTTCCCAACAGTTTCG), *dilp6*R (AAATCGGTTACGTTCTGCAAGTC), *Tub5F* (TCAGACCTCGAAATCGTAGC), *Tub5*R (GCCTGACCAACATGGATAGA), *dilp2*F (TCTGCAGTGAAAAGCTCAACGA), *dilp2*R (TCGGCACCGGGCATG), *rpL32*F (CGGATCGATATGCTAAGCTGT), *rpL32*F (GCCCTTGTTCGATCCGTA).

### Statistics

For courtship assay data, a Wilcoxon signed rank test was applied to test the null hypothesis of no preference (no difference from 50%), and Wilcoxon sum-rank test of t-test were used for pairwise comparisons. At least 3 replicates were run for each manipulation. Ovary size, fecundity, and expression levels data were analyzed with ANOVA followed by Tukey HSD. These analyses were done in Jmp 12 (SAS Institute Inc., Cary, NC), and the data were transformed before analysis where necessary to conform to ANOVA assumptions. All reported P-values are for 2-sided assumptions. Changes in CHC profiles upon insulin signaling activation and inhibition or *ovo*^*D1*^ introduction were assessed using Binomial sign test. To further describe CHC profile changes in multivariate space, we had also performed the principal component analysis on relative CHC abundances.

## Supporting information

S1 Fig IS manipulations in oenocytes do not affect attractiveness. For any duration of UAS transgene activation tested (6, 9, and 12 days of exposure to the activating temperature, 29°C) there was no effect on female attractiveness of IS manipulations in oenocytes when using *PromE800-Gal4*,*TubP-Gal80*^*ts*^ as a driver.
(EPS)

S2 Fig Systemic insulin signaling manipulations do not affect attractiveness. (A) Treatments known to affect systemic insulin signaling (Park et al. 2014) have no affect attractiveness, including manipulating insulin signaling (*gsDilp2>InR*, *gsDilp2>Pten*) or neuron firing activity (*Dilp2R>TrpA1*, *Dilp2R>Kir*^*2*.*1*^,*Tub-Gal80*^*ts*^*)* of brain insulin producing cells (IPCs), or female starvation (resulted in 10% mass loss). (B) *Dilp2* gene expression in the head is significantly increased by upregulation of insulin signaling in the late follicle cells of by its downregulation in the fat body.
(EPS)

S3 Fig Fecundity does not predict attractiveness. (A) Despite having the same (positive) effect on attractiveness (Fig 2B), the two opposing IS manipulations in early follicle cells had opposing effects on fecundity. (B) Upregulation of IS in oenocytes (using *PromE800-Gal4*, *tubP-Gal80*^*ts*^ as a driver) decreased fecundity and IS downregulation had no effect, while both manipulations did not affect attractiveness (S1 Fig).
(EPS)

S4 Fig CHC changes with ovarian ablation and with germline IS manipulations. (A) Ovarian ablation with *ovo*^*D1*^ either abolished or reversed individual CHC changes caused by global IS manipulations (compare to Fig 5B–these are part of the same experiment). All saturated CHC responses flipped for both IS manipulations (Binomial test for 7 out of 7, p = 0.0078). (B) The two opposing IS manipulations in the germline show no opposing effects on CHCs, corroborating the absence of their effect on attractiveness. No effect of IS manipulation on attractiveness is indicated by grey shading. For CHC identity refer to Fig 5.
(EPS)

S5 Fig CHC changes with oenocyte IS manipulations, and with ovarian ablation in Yolk>Pten females. (A) Saturated CHCs increased consistently in Yolk>Pten, the pattern reversed by ovarian ablation (Binomial test p = 0.0078). (B) IS manipulations in oenocytes changed CHC relative amounts independent of saturation class: although 20/28 total CHCs changed in opposite directions upon InR-OX vs Pten-OX manipulation (Binomial sign test P = 0.017), saturated CHCs did not all change in opposite direction for the two opposing IS manipulations (P = 0.226). No effect of IS manipulation on attractiveness is indicated by grey shading. For CHC identity refer to Fig 5.
(EPS)

S6 Fig Contrasting influence of tissue-specific IS on attractiveness is reflected in female CHC profiles. Principal component analysis of relative CHC abundances depicts desaturation changes (PC2) when insulin signaling is manipulated in the fat body and globally: proportion of saturated CHCs increase when insulin signaling is downregulated in the fat body (yellow signs, p = 0.0001), and this proportions decrease for the opposing IS manipulation (blue signs, p = 0.0005). Global downregulation of IS shows a trend toward an increased CHC desaturation (black filled symbols, p = 0.077), which is reversed upon introduction of *ovoD*^*1*^ (black open symbols, p = 0.002). Similarly, the direction of changes in CHC desaturation, though not quite statistically significant for downregulation of IS when compared to UAS control (olive filled symbols), reverses in direction for PC2 when *ovo*^*D1*^ is introduced (olive open symbols, p = 0.034). PC1 best separates genetic backgrounds and temperature treatment (germline & late FC), and reflects CHC elongation changes upon transgenic manipulations. (A) Ellipses delineate specific tissue Gal4 drivers and addition of *ovo*^*D1*^; same color signs indicate comparisons within the same genetic background; diamond signs indicate control treatments, triangles pointing up indicate flies with *InR* overexpression, and triangles pointing down are flies with *Pten* overexpression. (B) Loading scores after Quatrimin rotation of 2 retained principal components explaining 78% total variation in CHCs. Factor loadings preferentially loading on one of the 2 PCs (= more than 0.6 for one PC and less than 0.4 for the other) are marked in bold, and known saturated CHCs are shown in red font. PC1 seem to separate genetic backgrounds as well as abundance of longer versus shorter CHCs, while PC2 is more indicative of the abundance of saturated versus unsaturated CHCs.
(EPS)

S7 Fig Model for tissue insulin signaling effects on female attractiveness. Schematic representation of the effects observed in this study of tissue-specific insulin signaling on female attractiveness (solid blue lines) and on tissue *dilp2 or dilp6* mRNA (dashed blue lines). Black suppression arrow is based on Bai et al. (2012). We have not detected any effects of oenocyte, germline, early follicle cell, or systemic (IPC) IS manipulations on attractiveness.
(EPS)

S1 File CHC abundances upon manipulation of insulin signaling in various tissues. (XLS)
